# The evolution of the 2022–2024 eruption at Home Reef, Tonga, analyzed from space shows vent migration due to erosion

**DOI:** 10.1038/s41598-025-95197-2

**Published:** 2025-04-03

**Authors:** Simon Plank, Emanuele Ciancia, Nicola Genzano, Alfredo Falconieri, Sandro Martinis, Hannes Taubenböck, Nicola Pergola, Francesco Marchese

**Affiliations:** 1 German Aerospace Center DLR, German Remote Sensing Data Center, 82234 Oberpfaffenhofen, Germany; 2 Institute of Methodologies for Environmental Analysis, National Research Council, 85050 Potenza, Tito Scalo Italy; 3 Department ABC (Architecture, Built Environment and Construction Engineering), Politecnico di Milano, via Ponzio 31, 20133 Milano, Italy; 4 Space Technologies and Application Center, University of Basilicata, 85100 Potenza, Italy; 5 Earth Observation Research Cluster, University of Würzburg, 97074 Wurzburg, Germany

**Keywords:** Island forming volcanism, Erosion controls vent migration, Multi-sensor satellite volcano monitoring., Volcanology, Natural hazards

## Abstract

On September 9, 2022, a new eruption period began at the submarine volcano Home Reef, part of the Tonga Volcanic Arc. We integrated multi-sensor/multi-platform satellite datasets, including very high spatial resolution TerraSAR-X radar and PlanetScope multispectral data, together with Sentinel-2 and Landsat-8/9 as well as MODIS and VIIRS thermal data to monitor and characterize this latest eruption at Home Reef over a two-year period. Here, we present the results from this multi-sensor approach, used to investigate eruption dynamics (thermal activity and relative intensity level) and delineate changes in the shape and area of the newly formed island. The eruption showed four distinct phases: During September–October 2022, lava flows formed a ~ 54,900 m² circular island. In the following three eruption phases, the island grew towards the south (September–November 2023) and east (January 2024 and June–September 2024), expanding the island’s area to over 122,000 m². During each subsequent phase, the eruptive vent migrated toward the side of the island where the most erosion had occurred since the previous phase. This has implications for volcanic and tsunami hazards from island-forming eruptions of this type.

## Introduction

The Hunga Tonga-Hunga Ha’apai (Tonga) volcano-tsunami event of January 15, 2022, has shown the catastrophic impact submarine volcanoes can have on coastal areas located at considerable distances from the source^1–3^. This was one of the most powerful volcanic eruptions recorded in recent history (the strongest since the eruption of Mt. Pinatubo in 1991)^4^. Besides the catastrophic local tsunami affecting the neighboring islands of Hunga Volcano, the accompanying volcano-meteorological tsunami also had global effects: the tsunami hit the coasts of Japan and Peru, for example, and in the latter case a discharge ship was rocked by the tsunami waves causing an oil spill; smaller tsunami waves were even measured in the Caribbean and Mediterranean Sea. These events triggered activations of the International Charter “Space and Major Disasters” to support rapid damage mapping activities^5,6^. This event showed the importance of monitoring volcanoes even in very remote areas as their eruptions may have regional and even global impacts.

Satellite observations can be exploited to investigate and monitor the surface effects of submarine eruptions through the analysis of thermal activity, estimates of total erupted material, and the characterization of both growth and erosion phases of newly formed islands^7–9^. In this context, MODIS (Moderate Resolution Imaging Spectroradiometer), VIIRS (Visible Infrared Imaging Radiometer Suite), Landsat (L8/9) Operational Land Imager (OLI/OLI-2) and Sentinel-2 (S2) Multispectral Instrument (MSI) sensors, by providing data from visible to infrared data at different spatial resolution, have already demonstrated their capability to contribute to the monitoring of remote volcanoes^10,11^. Integration with information retrieved from Synthetic Aperture Radar (SAR) data may enable an even more effective monitoring of volcanic activity in remote and inaccessible areas^8,12^.

In this study, we present the results of a comprehensive multi-platform and multi-sensor satellite monitoring effort of the Home Reef, Tonga, an island forming volcano, investigating recent eruption phases in 2022–2024. Data retrieved from a multi-sensor dataset of satellite observations (i.e., S2, L8/9, MODIS, VIIRS, PlanetScope (PS), TerraSAR-X (TSX)) are used to monitor and quantify the surface volcanic activity and to characterize the newly formed island. For each of the four eruption phases, we observed that the active vent was in a different location. Our analysis suggests that erosion of the newly formed volcanic island was a key factor for the position of the vent in subsequent phases.

### Regional setting and recent volcanic eruptions in Tonga

This study investigates island forming volcanism in one of the most active volcanic arcs on Earth: the Tonga Volcanic Arc, which is located west of the Tonga Trench in the South Pacific. The Tonga Islands form the northern end of an island arc system that extends discontinuously from south of Samoa SSW towards New Zealand^13^.

**Fig. 1:**
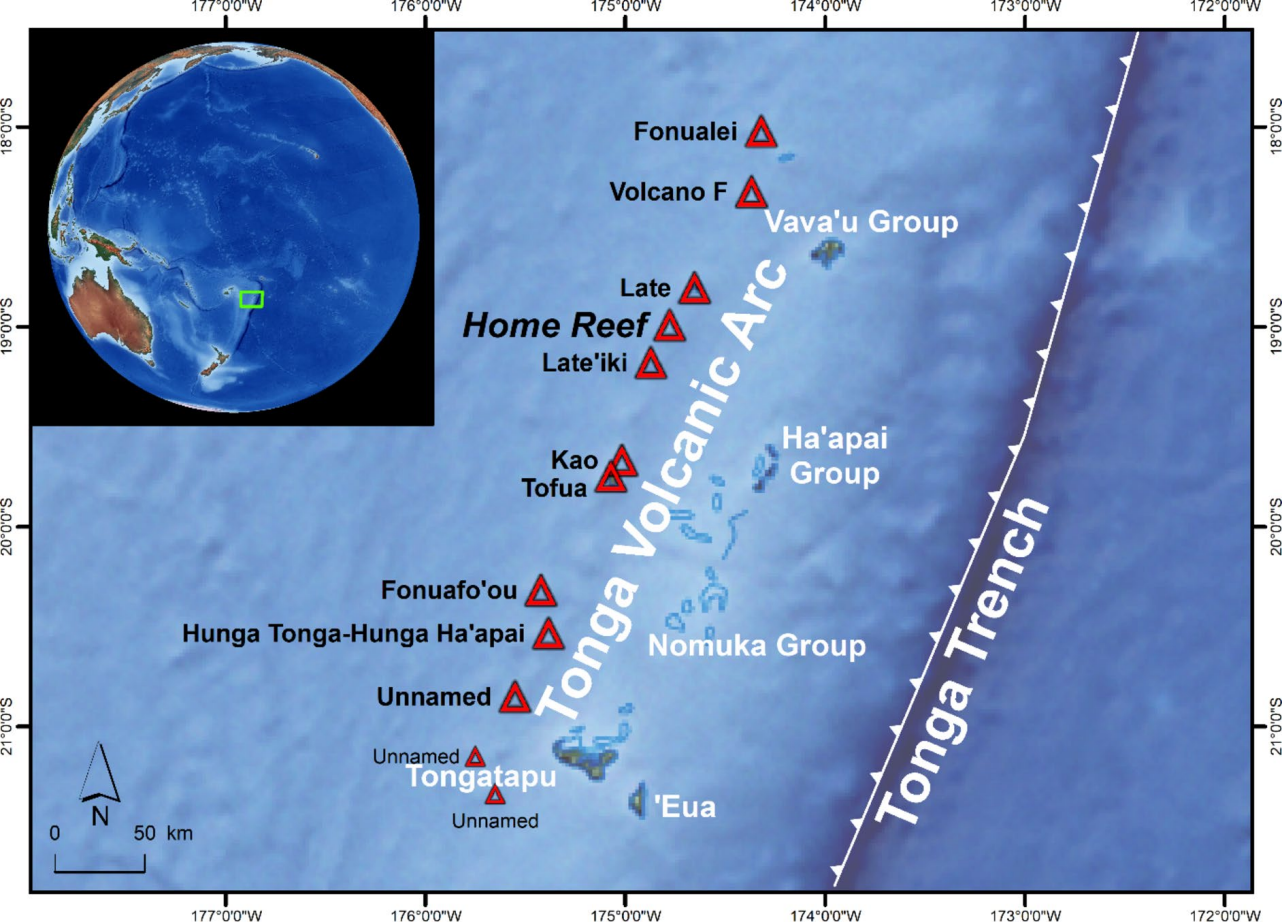
Home Reef is one of several submarine and island volcanoes (red triangles) located on the Tonga Volcanic Arc, west of the Tonga Trench in the South Pacific. The green rectangle on the inset map marks the location of the more detailed map. Map modified after^8,16,17^. Background: Made with Natural Earth (naturalearthdata.com). Map created using ESRI ArcGIS Pro 3.3 (https://www.esri.com/en-us/arcgis/products/arcgis-pro).

The powerful volcanic eruption at Hunga Tonga-Hunga Ha’api in January 2022 made the area famous, but less well-known are a series of much less powerful eruptions that have recently occurred nearby (Fig. 1). Examples of this submarine volcanic activity include the eruption of an unnamed volcano in January 2017 (listed as #243030 in the database of the Global Volcanism Program GVP^14^ approx. 37 km SW of Hunga Tonga-Hunga Ha’api, forming a 30 km long and 20 km wide plume of discolored water, or the eruption of Volcano F in August 2019 (GVP database #243091) located approx. 60 km N of Late Island, creating extensive areas of pumice rafts^15^. A recent example of island forming volcanic activity is the eruption at Late’iki Volcano in 2019, located 47 km SSW of Late Island, where the remains of an island that formed in 1995 were destroyed and a new, but short-lived island was formed in its place^8^. At Home Reef Volcano (18.992°S, 174.775°W), located halfway between Late’iki and Late Island, a new island was formed during an eruption period in September–October 2022. Later, this island was expanded during a second eruption phase occurring in September–November 2023, followed by a third and fourth phase in January and June−September 2024. The formation and temporal evolution of this youngest island of the Tonga Volcanic Arc is the topic of this article.

### The history of volcanic activity and island formation at Home Reef

The GVP^18^ database lists four confirmed eruption events at Home Reef Volcano during which ephemeral islands were produced: in 1852, in March 1984, in August 2006 and in September–October 2022 and continued in September–November 2023 and into 2024. The following gives a summary of the GVP reports for the activity in 1984 and 2006^18^.

On March 1–5, 1984, during an intense submarine eruption, Home Reef Volcano built two small islands with a maximum elevation of about 20 to 50 m, enclosing a crater. The total area of the island was about 1,500 m × 500 m.

The next reported eruption period was observed in MODIS imagery for the first time on August 7, 2006. Five days later, a yacht crew reported an island of ~ 1.77 km². The island eroded rapidly. On October 4, 2006, a first High Resolution (HR) satellite observation by ASTER (Advanced Spaceborne Thermal Emission and Reflection Radiometer) showed an island that was only ~ 0.24 km² in area. The last observation of the island with an area of only ~ 0.003 km² was by Landsat-7 on April 7, 2007. Field investigations in November 2008 reported that the island was completely eroded to about 9–10 m below sea level.

### Satellite observations for monitoring volcanic activity in remote areas

Satellite-based Earth observation allows for the detection, monitoring and analysis of active volcanoes on a global scale^19^ and it is helpful for investigating volcanoes in remote and inaccessible areas where there is no in-situ monitoring network installed. The combination of thermal, optical and SAR satellite sensors allows for monitoring and investigation of various aspects of volcanic activity. The specific satellite data used in this study are described in detail in the Materials and Methods section (Table 1, Sect. 5) at the end of this paper. Below, we provide a shorter summary of the data we utilized.

Infrared (IR) sensors on geostationary satellites Meteosat, GOES (Geostationary Operational Environmental Satellite) or Himawari are used in automated systems for near real time analysis of volcanic activity: e.g. RST_VOLC_^20^, HOTSAT^21^ or HOTVOLC^22^. Geostationary IR data can provide precise timing of the onset of an eruption^23^. Constellations of mid-resolution IR sensors such as MODIS, VIIRS, SLSTR (Sea and Land Surface Temperature Radiometer), operating on polar-orbiting satellites, provide a good compromise between a spatial resolution (between several 100 m and ~ 1 km) high enough for detection of volcanic thermal anomalies and at an observation frequency (several observations per day) suitable for monitoring the evolution of volcanic activity. MODVOLC and MIROVA (Middle Infrared Observation of Volcanic Activity) are examples of automated volcanic hotspot detection systems using these mid-resolution data^10,24^. Timeseries analysis of mid-resolution IR sensors has been used by others to estimate discharge rates and volumes of lava flow events, for example for the 2014/15 Holuhraun fissure eruption in Iceland^25^, the 2018 eruption of Kīlauea, Hawaiʻi^26^ and the 2021 Cumbre Vieja eruption in La Palma^27^. Detailed thermal analysis of lava flows^23^, lava domes^28^ and lava lakes require higher spatial resolution short wave IR (SWIR) data with a resolution in the order of 10s of meters as provided by the Landsat-5/7/8/9 and Sentinel-2 satellites. However, the longer repeat cycles (5–16 days) of these satellites do not allow for the analysis of short-term changes as is possible with satellites having mid-resolution sensors onboard^27^ (cf. Table 1 in Sect. 5.1, below).

Satellites with HR and very high spatial resolution (VHR) optical sensors, such as PlanetScope, Pléiades, WorldView, enable detailed analysis of the emplacement of pyroclastic density currents and lava flows^29^. The latter two satellite missions have stereo-imaging capabilities to generate digital surface models for detailed 3-dimensional investigation of the structures and volumes of lava domes and lava flows^30^. While these stereo images can theoretically be produced as often as every several days over a specific area of interest, the high number of small Cubesat satellites (currently about 130) of the PlanetScope constellation^31^ allows a daily coverage of anywhere on the Earth’s landmass. Due to its high observation frequency, timeseries of PlanetScope data were used, for example, for rapid deposit mapping at Fuego Volcano (Guatemala)^32^ or for near real-time mapping of tephra fallout at Mt. Etna (Italy)^33^. Of course, clear sky conditions are required for usable images. A major limitation regarding the monitoring applications of volcanic activity is the high cost of these commercial VHR data.

In contrast to the optical and thermal satellite sensors mentioned above, SAR is the only satellite sensor type that provides useful data during cloudy conditions, be it a volcanic ash plume or a meteorological cloud. Interferometric SAR analysis enables the measurement of small deformation (cm–dm scale) of a volcano’s surface related to magma chamber inflation or deflation, propagation of a dyke or movement of volcanic flanks^34^ – provided that the interferometric coherence stays high enough between the satellite data acquisitions. SAR amplitude analysis also can provide information about the volcano surface when major changes occur, e.g., caused by explosive eruptions^35^.

Using a combination of different satellite sensor types is an approach that overcomes their individual limitations and yields a more comprehensive understanding of complex volcanic processes^19^. Such a multi-sensor approach was exploited, for example, for the investigation of the 2018 flank collapse of Anak Krakatau in Indonesia^36^ and the 2021 eruption of Cumbre Vieja in La Palma^27^.

## Results

In this study, we focus on the analysis of satellite data for investigating the processes of island formation and erosion during the latest eruption period in 2022–2024 at Home Reef Volcano. The satellite data acquired and analyzed over the Home Reef Volcano area are listed in Table 1 (Sect. 5.1, below). This analysis shows that this eruption was characterized by four phases of activity: (1) A *first phase* from September 9, 2022, until beginning of October 2022. (2) A *second phase* in September–November 2023, followed by (3) a *third phase* in January 2024 and a (4) *fourth phase* between June–September 2024.

### First eruption phase (September–October 2022)

**Fig. 2:**
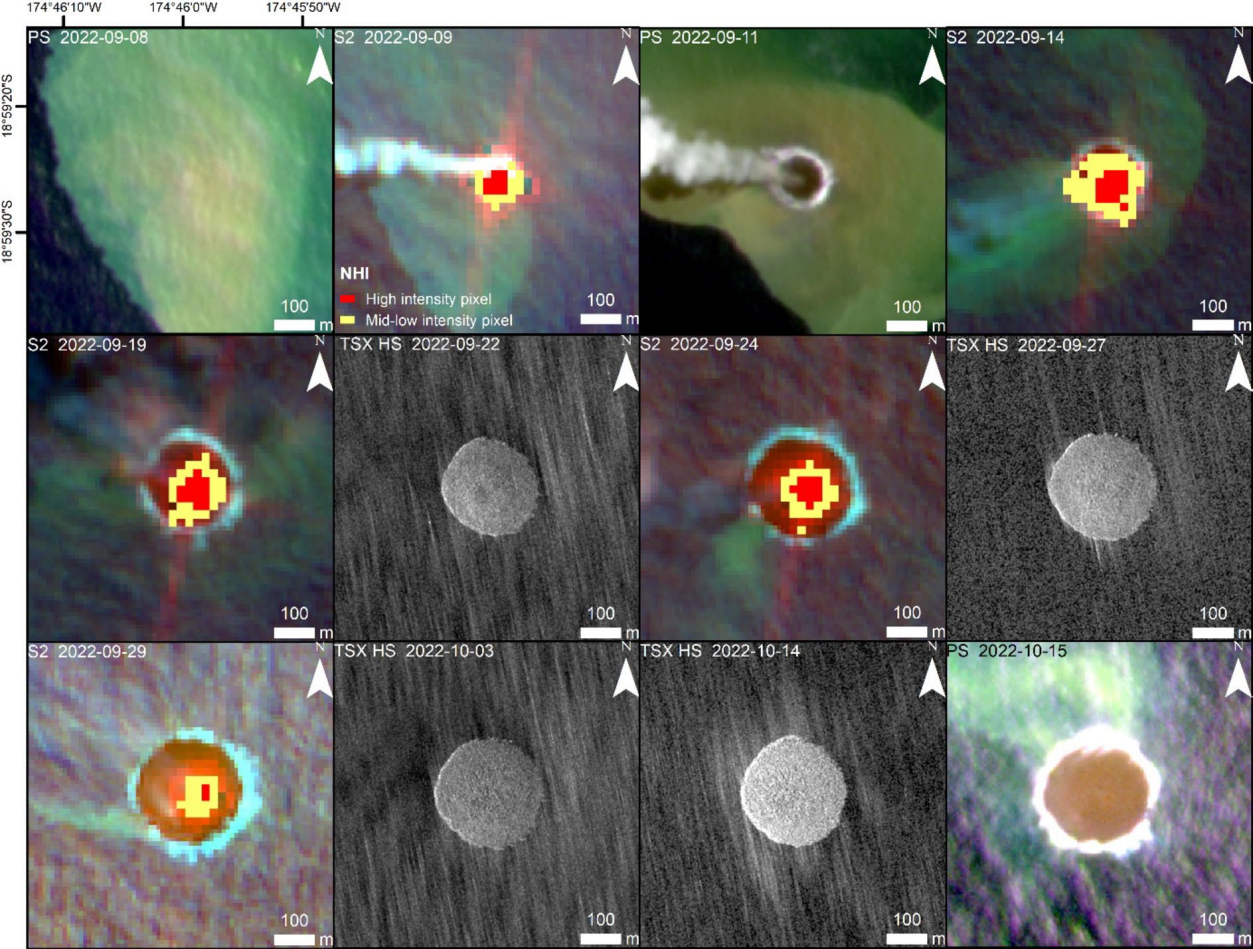
Island formation at Home Reef Volcano during the initial eruption phase in September 2022, monitored by PS (true color 6/4/2), TSX-HS and S2 (false color composite 12/4/2). Sensor type and acquisition date is in the upper left of each image and scale is in the lower right. The first image shows discolored water from strong submarine volcanic activity one day before the volcanic island began to grow above the sea surface. Based on the NHI analysis of S2 data, high thermal emissions are shown in red, mid-low thermal emissions in yellow. The thermal emissions were concentrated at the center of the newly formed island. Satellite imagery: PlanetScope (Planet Labs 2022), TerraSAR-X/TanDEM-X DLR e.V. (2022), Sentinel-2 Copernicus data (2022). Map created using ESRI ArcGIS Pro 3.3 (https://www.esri.com/en-us/arcgis/products/arcgis-pro).

**Fig. 3:**
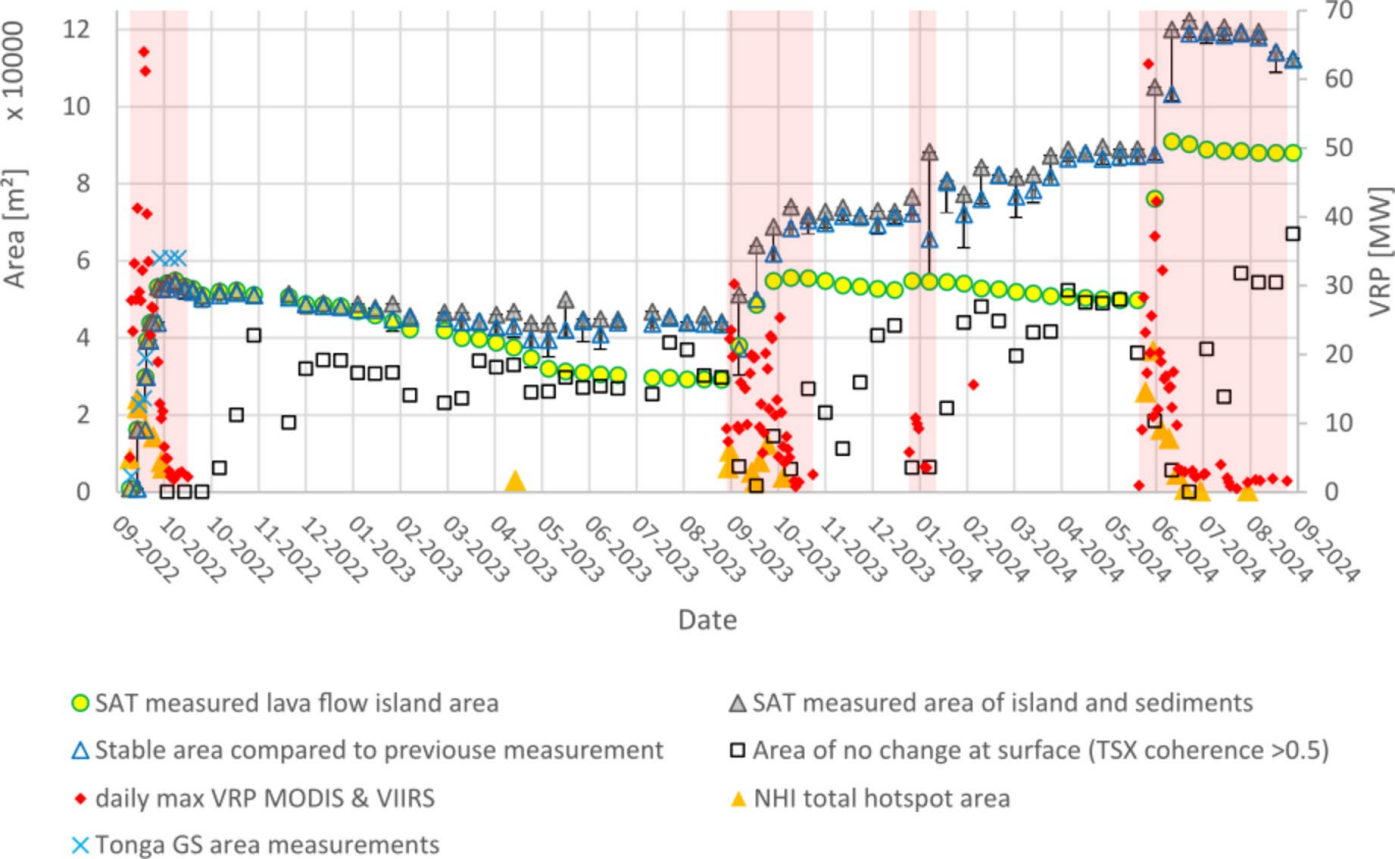
Island growth and erosion at Home Reef Volcano in 2022–2024. TSX-HS satellite (SAT) data-based area measurements of the lava flow island (green-yellow circles). Area of the entire island (lava flows and sediments) (grey filled triangles). Island (lava flows and sediments) area stable compared to the previous measurement (open blue triangle). Loss due to erosion compared to the previous measurement shown by black line downwards from the blue triangle. Newly grown island area compared to the previous measurement shown by black line upwards from the blue triangle. In the stable phases between the four eruption phases, this is due to accumulation of volcanic sediments that previously eroded from the lava flow island. Island area with no change on the surface based on TSX interferometric coherence (> 0.5) comparing consecutive acquisitions (black squares). Cyan crosses represent the area measurements according to the Tonga Geological Survey (TGS)^38^. Orange triangles show the area of high thermal emission monitored by S2 and L8/9 SWIR data based on the NHI. Red diamonds show the maximum VRP (Volcanic Radiative Power) per day monitored by MODIS or VIIRS. Duration of eruption phases are marked in pink shading.

On September 9, 2022, thermal volcanic activity at Home Reef was recorded for the first time in satellite imagery (Figs. 2 and 3). On that day the NHI (Normalized Hotspot Indices) system, which analyzes S2 and L8/9 NIR and SWIR band data (discussed in more detail in Table 1 and in Sect. 5.2.2, below), automatically signaled the beginning of new volcanic activity above the sea surface and the formation of a new island at Home Reef.

Figure 2 shows a PS image acquired just one day before the island began to grow above the sea surface. A large plume of discolored water is clearly visible. The island grew dramatically in size and volume until September 27, 2022, with a growth rate of ~ 2955 m²/day as measured by S2 and TSX HighResolution SpotLight (HS) satellite imagery. Then, the growth rate slowed down to ~ 170 m²/day. The maximum island area in the first eruption phase of ~ 54,900 m² was measured on October 8, 2022.

According to analysis of S2 data, the area of high thermal emission increased from September 9, 2022 and reached its maximum on September 14, 2022 (Figs. 2 and 3). Declining thermal activity was observed by the end of the month.

Daily maximum measurements of Volcanic Radiative Power (VRP), observed by the thermal sensors MODIS and VIIRS (NASA FIRMS^37^, cf. also Table 1 in Sect. 5.1, below), show similar results to the NHI S2 and L8/9 analysis. The maximum VRP of 64 MW was observed on September 18, 2022. Thermal emissions of < 5 MW were observed from October 2 onwards, marking the end of the first eruption phase (Fig. 3).

After the end of the first eruption phase (beginning of October 2022), the area of the island remained relatively stable (Fig. 3). From February until the end of July 2023, erosion by ocean waves continuously decreased the area of the original lava flow island (yellow-green circles in Fig. 3) by ~ 110 m²/day. The erosion rate of the original island declined to ~ 15 m²/day during the next three months. From the end of May until the beginning of July 2023, the entire island’s perimeter showed changes due to increased erosion effects and the accumulation of sediments previously eroded from the island. Despite that, the island area remained relatively stable. The island’s center showed very little change as shown by high values (> 0.5) of the interferometric coherence of consecutive TSX-HS acquisitions between December 30, 2022, and September 20, 2023. The interferometric coherence describes the phase stability between two consecutive SAR acquisitions and is more sensitive to small changes on the surface compared to SAR backscatter analysis (cf. Section 5.2.1).

The absence of other significant thermal anomalies at Home Reef during this period, except from a small hotspot automatically detected by the NHI system on the S2 scene of May 12, 2023, is consistent with this evidence.

### Second eruption phase (September–November 2023)

After this quiet period, increasing thermal activity was again detected by the NHI from S2 data on September 14, 2023 (Fig. 3). The second eruptive phase appeared less intense than the previous one, as indicated by the maximum value of daily VRP, although its duration was longer.

**Fig. 4:**
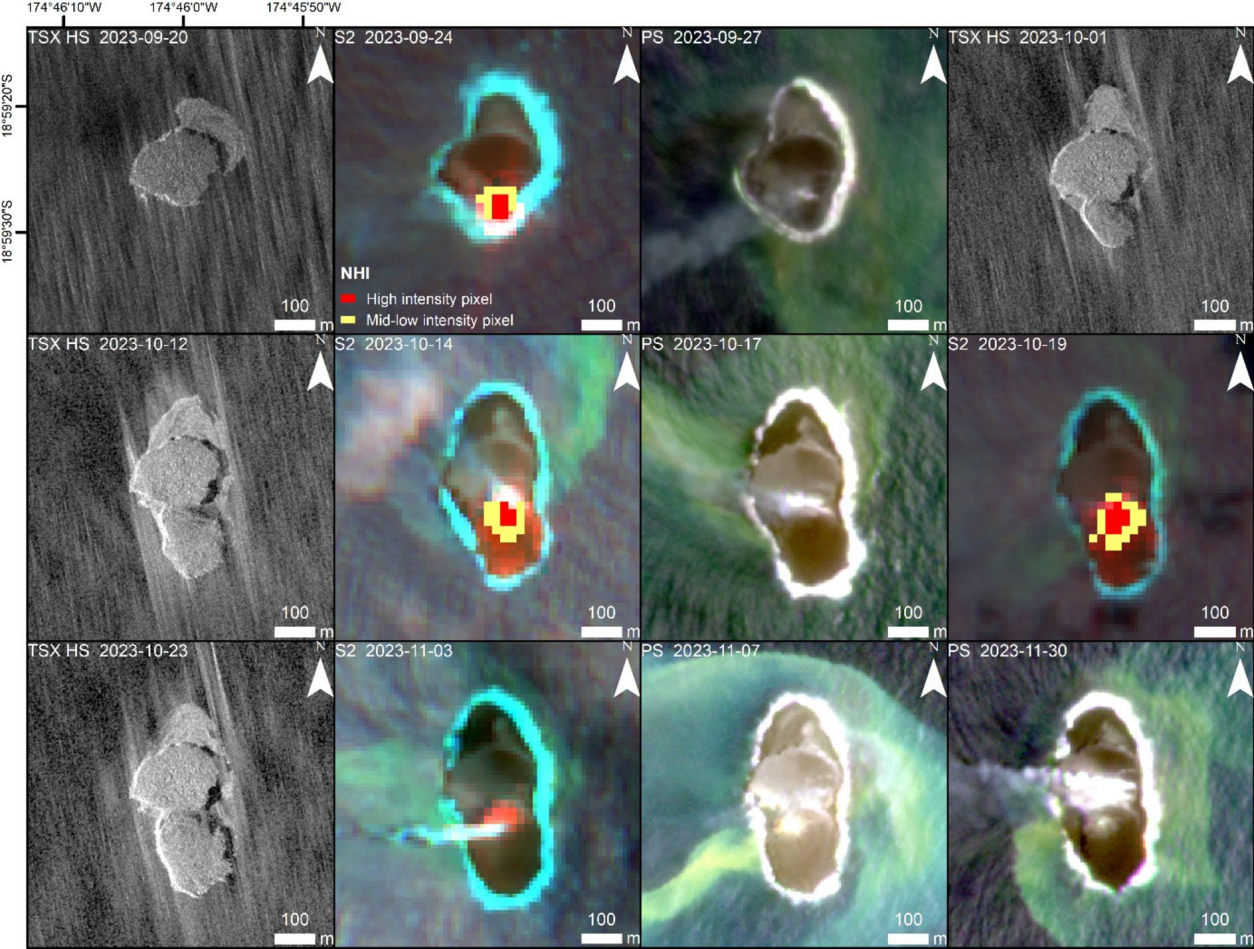
Expansion of Home Reef Island during the second eruption phase in September-November 2023 due to a lava flow at the southern edge of the island formed during the first eruption phase (cf. Figure 2). On November 3, 2023, although visible in the SWIR data, the thermal signal already was too weak to be detected by the standard NHI algorithm. PS and S2 data showed plumes of discolored water. Satellite imagery: PlanetScope (Planet Labs 2023), TerraSAR-X/TanDEM-X DLR e.V. (2023), Sentinel-2 Copernicus data (2023). Map created using ESRI ArcGIS Pro 3.3 (https://www.esri.com/en-us/arcgis/products/arcgis-pro)

Figure 4 shows thermal anomalies observed by S2 on the southern side of the island consistent with the location of white water vapor plumes visible in the PS data. The subsequent new lava flow in the southern direction is visible in the TSX-HS data. From September 20 until November 3, 2023, the island area grew by ~ 677 m²/day up to 74,000 m² (Fig. 3). Plumes of discolored water visible in the S2 and PS imagery showed ongoing submarine volcanic activity during this second eruption phase.

### Third eruption phase (January 2024)

**Fig. 5:**
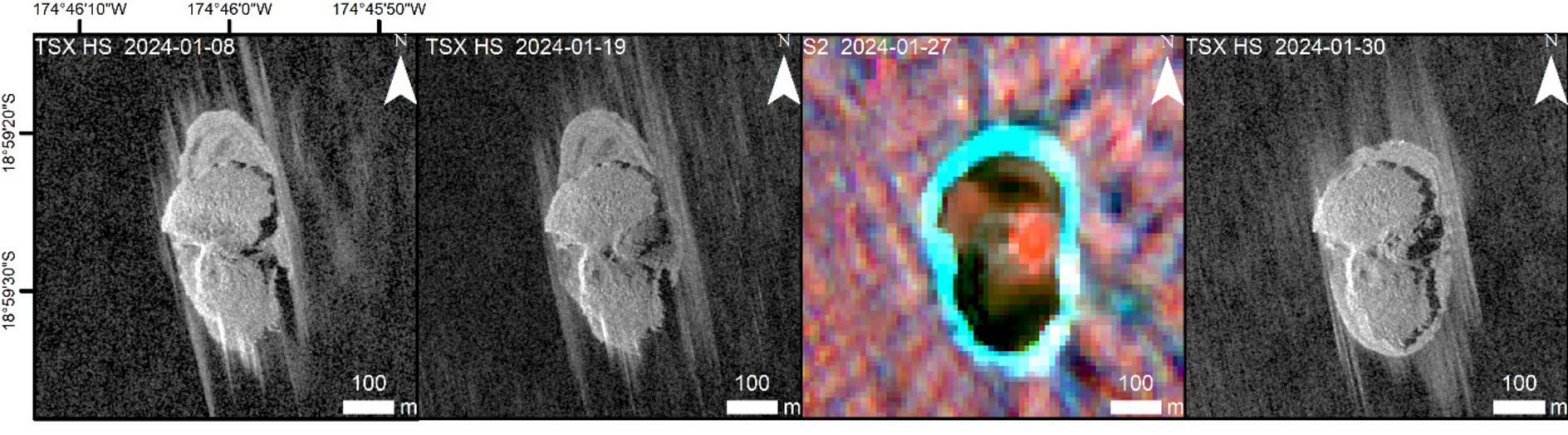
Lava flow to the east during the third eruption phase in January 2024. On January 27, 2024, although visible in the SWIR data, the thermal signal already was too weak to be detected by the standard NHI algorithm, so may represent lava cooling. Satellite imagery: TerraSAR-X/TanDEM-X DLR e.V. (2024), Sentinel-2 Copernicus data (2024). Map created using ESRI ArcGIS Pro 3.3 (https://www.esri.com/en-us/arcgis/products/arcgis-pro).

In Fig. 3 VRP measurements on January 17, 2024, show the beginning of a short-lived (11 days) and relatively weak (max VRP 10.7 MW) eruption phase, during which the island was expanded by ~ 2,400 m² towards the east (Fig. 5). During February 2024, one thermal signature captured by MODIS and VIIRS and low coherence values suggest there may have been low-level activity continuing, but the lava flow island area remained stable.

From March 2024 until mid-June 2024, we observed a decrease of the lava flow island area by ~ 39 m²/day, while the area of the entire island slightly increased due to accumulation of eroded material.

### Fourth eruption phase (June–September 2024)

**Fig. 6:**
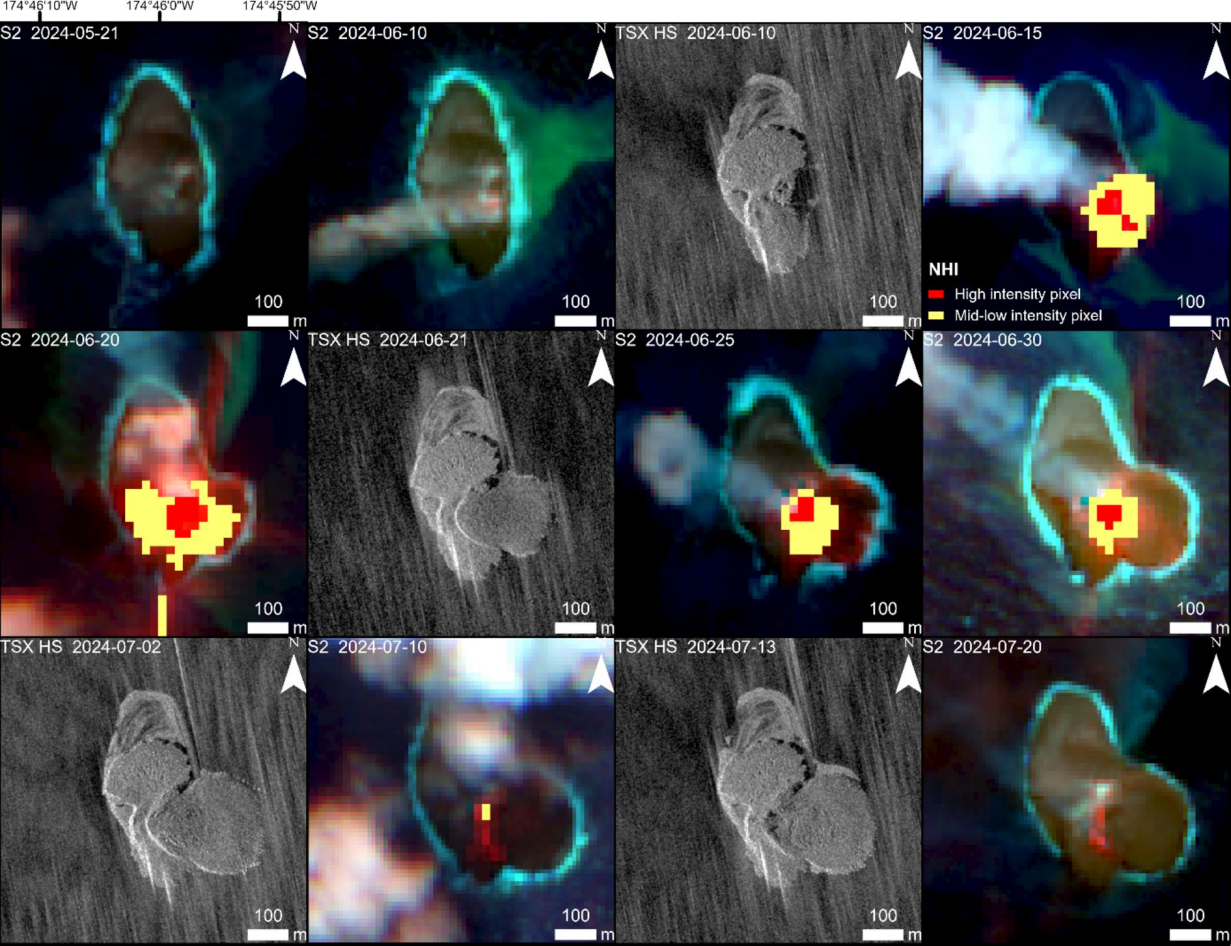
Large lava flow towards the east during the fourth eruption phase in July–September 2024. Satellite imagery: TerraSAR-X/TanDEM-X DLR e.V. (2024), Sentinel-2 Copernicus data (2024). Map created using ESRI ArcGIS Pro 3.3 (https://www.esri.com/en-us/arcgis/products/arcgis-pro).

On June 11, 2024 the fourth eruption phase began. It was characterized by a large lava flow towards the east. Until July 2, 2024, the lava flow island area almost doubled to 90,900 m², with a growth rate of 1,870 m²/day (Figs. 3 and 6). The intense period of VRP > 10 MW was three weeks long. The area of the entire island increased from ~ 87,000 to over 122,000 m². The duration and maximum thermal values during the fourth phase (max. VRP > 60 MW) were comparable to the first one in 2022. However, the waning phase (with VRP < 5 MW) of the fourth eruption phase was four times longer compared to the first phase.

## Discussion

### Erosion controls vent migration

**Fig. 7:**
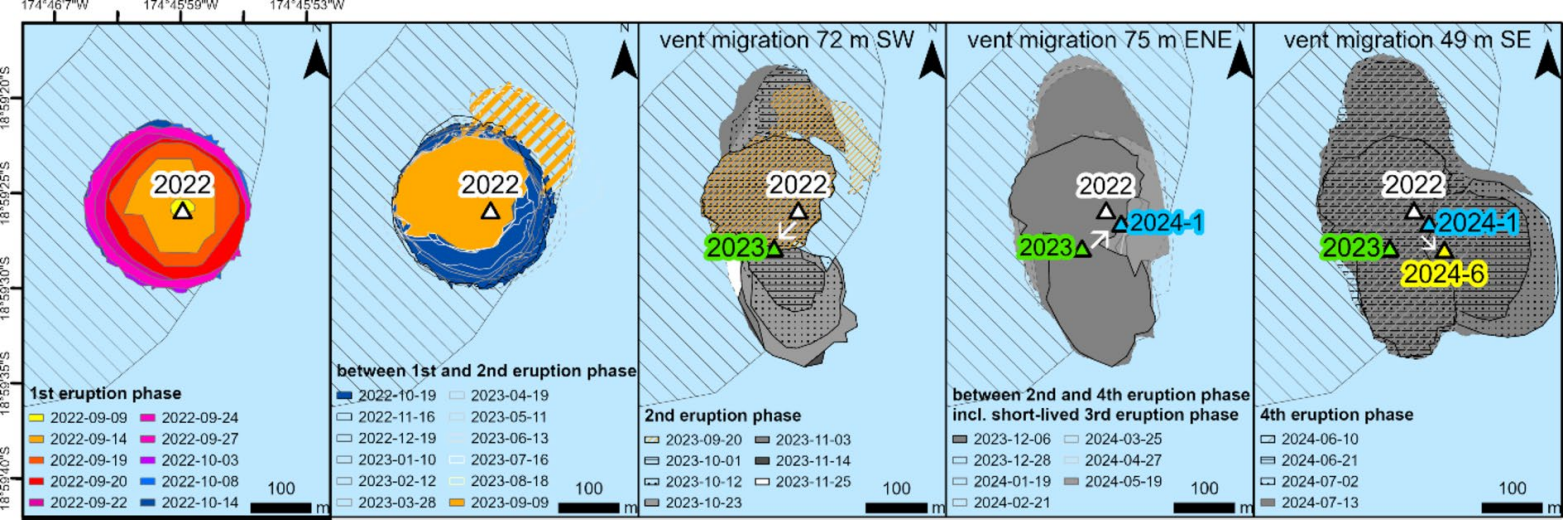
From left to right: (1) Island growth during the first eruption phase (September–October 2022); (2) island erosion and accumulation of sediments in the north (between the first and second eruption phase); (3) island growth to the south during second eruption phase (September–November 2023); (4) small island expansion to the east during third eruption phase (January 2024); (5) large island expansion during fourth eruption phase (June–September 2024). The triangles mark the location of the active vents. The white arrows show the vent migrations. Solid outlines show the lava flow island, dashed outlines the sediments. The aerial extent of the island formed in 2006 as observed by ASTER on October 4, 2006 is shown in stripped grey. Map created using ESRI ArcGIS Pro 3.3 (https://www.esri.com/en-us/arcgis/products/arcgis-pro).

During the first eruption phase, a circular island built up of lava flows grew on top of the remnants of the 2006 Home Reef Island, which were at about 9–10 m below sea level according to observations in November 2008. The majority of the 2022–2024 island formed within the area of the 2006 island, based on the first available ASTER HR satellite image, acquired on October 4, 2006, three months after the 2006 eruption (Fig. 7). All the 2022–2024 vents are also located within the area of the 2006 island in the ASTER image. However, by the time of the ASTER image the island had already eroded considerably. The island that formed in 2006 was originally about seven times larger, based on observations from a yacht five days after the eruption onset, but we have no information about the extent of the original 2006 island towards the east where the latest flow was observed in 2024.

**Fig. 8:**
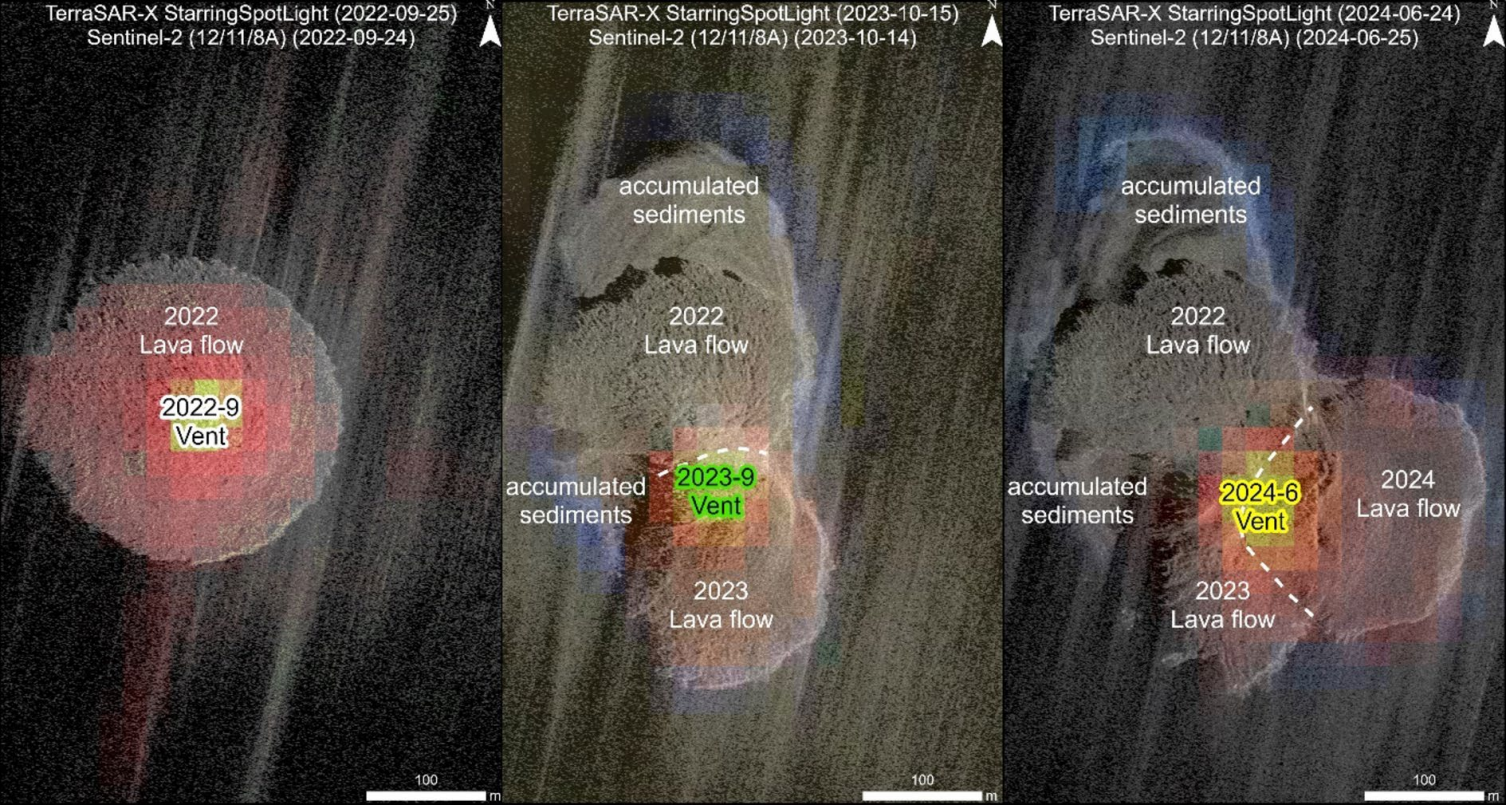
Examples of VHR TSX Starring SpotLight (ST, 25 cm resolution) images showing details of the island formation during the initial eruption phase (left image: acquisition dates September 24 and 25, 2022), the second island growth (middle image: acquisition dates October 14 and 15, 2023) and the island growth to the east during the fourth eruption phase (right image: acquisition dates June 24 and 25, 2024). The white dashed lines mark the border between the partly eroded 2022 lava flow island and the new lava flows of 2023 and 2024, respectively. Thermal information shown by the overlaid S2 data (false color composite 12/11/8A) shows a migration of the vents towards the south and east, respectively. The third eruption phase is not shown here due to its comparatively low level of activity (cf. Figures 3 and 5). Satellite imagery: TerraSAR-X/TanDEM-X DLR e.V. (2024), Sentinel-2 Copernicus data (2024). Map created using ESRI ArcGIS Pro 3.3 (https://www.esri.com/en-us/arcgis/products/arcgis-pro).

The 2022 lava flow island grew above sea level on September 9, 2022 from a vent located at the center, and reached its maximum size of ~ 54,900 m² on October 8, 2022 (Fig. 3). After the end of the first eruption phase, ocean waves eroded the island’s coast and formed steep cliffs. The main erosion took place in the southern and south-eastern part of the island. Eroded material accumulated near the shoreline and formed temporary “beaches” (Figs. 4 and 8).

**Fig. 9:**
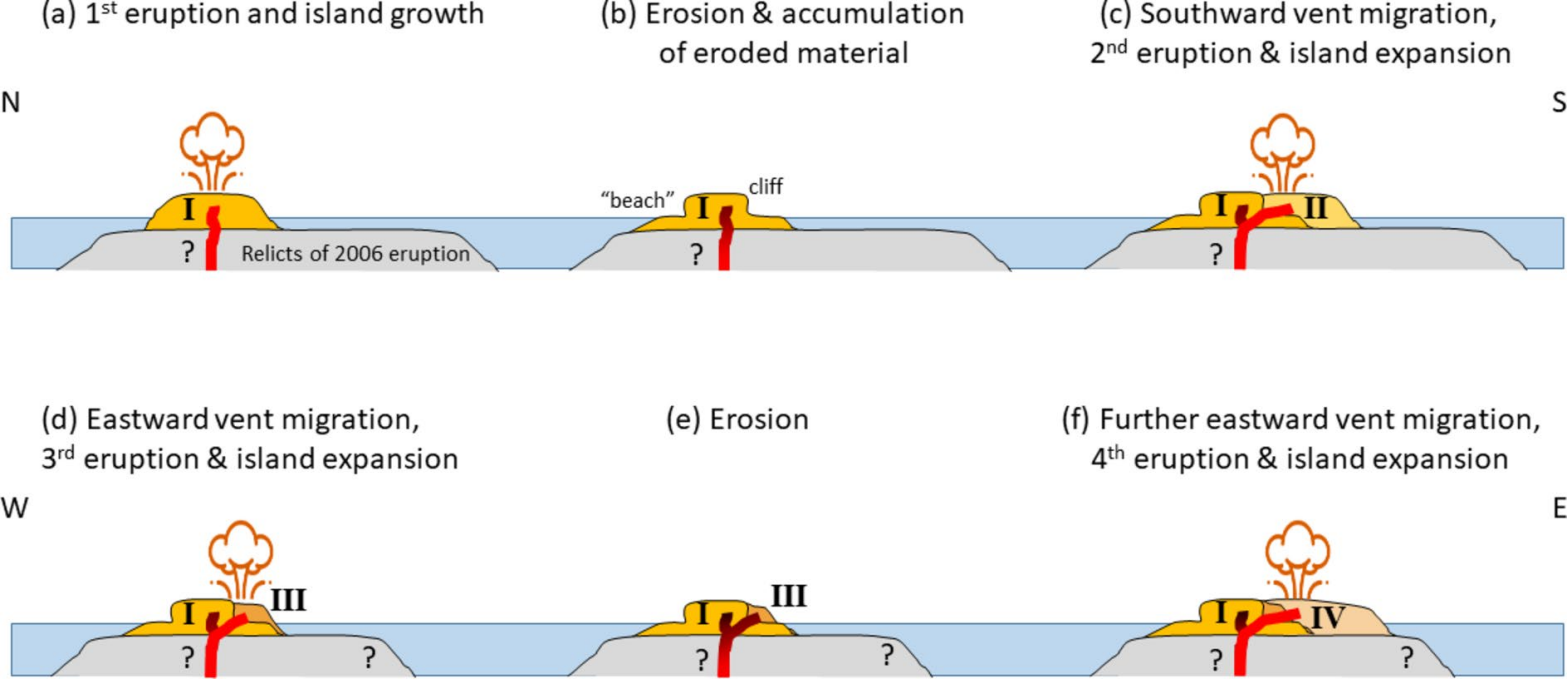
First row: North-South cross section of an evolutionary model of the 2022–2024 Home Reef Volcano eruption. (a) First eruption phase September–October 2022: Island forming lava flows on top of the remnants of the 2006 island. (b) Island erosion and accumulation of eroded material (“beach” formation) between the first and second eruption phase. (c) Second eruption phase September–November 2023: Vent migration towards the south and second lava flow event (II). Second row: West-East cross section showing (d) the lava flow and island expansion during the third (III) eruption phase in January 2024, the (e) erosion following at the east and (f) the further island expansion and eastward vent migration during the fourth eruption phase (IV).

During the second eruption phase (September–November 2023), a second lava flow expanded the island towards the south (Fig. 9). Satellite imagery from September 24, 2023 (Fig. 4) shows that the 2023 vent was located at the southern edge of the remnants of the original island formed in 2022. Compared to the vent in 2022, the 2023 vent had migrated about 72 m towards the south-west (Fig. 7) at the location where the strongest erosion occurred during the months before. We interpret that the removal of the load on that side, due to the partial erosion of the 2022 lava island, caused the vent to migrate in that direction. Magma takes the path of least resistance, opening along least compressive stress, so any local change in the stress field from overburden of new lava could affect vent migration like observed at Stromboli by Schmid et al.^39^. Vent migration on much longer time scales of several decades have been described, for example, for Bezymianny Volcano, Kamchatka^40^. Moreover, lateral collapses have the potential to change the location of the active vent, as described (on larger scales than Home Reef) by Maccaferri et al.^41^.

The vent active during the third eruption phase in January 2024, formed directly at the eastern edge of the 2022/23 island (75 m east-north-east of the 2023 vent). Again, the new vent formed where the strongest erosion had occurred during the months before. After the January 2024 eruption, erosion of the lava flow island mainly took place south and south-east of the January 2024 vent. Then in June 2024 the same pattern continued with the next new vent forming (49 m south-east of the January 2024 vent) where erosion had been strongest during the previous inter-eruption time interval. During this fourth eruption, the island grew strongly towards the east (Fig. 9).

The stability of volcanic edifices is reduced by structural and lithological discontinuities, magmatic intrusions, and high lava accumulation rates^42^ (like the rapid lava flow island growth in 2022–2024 at Home Reef). Volcanic islands often grow on weak substrata, which also reduces their stability^43^. The sequence of events during the 2022–2024 eruption supports a model in which the location of new eruptive vents is strongly influenced by where marine erosion had previously been most concentrated, which also influenced the direction in which the new lava flows subsequently grew.

This model has implications for volcanic hazards from island-forming eruptions. The areas to which the lava flows are directed may also influence where slope instability could lead to landslides, which could in turn trigger a local tsunami. On the other hand, it is important to note that due to its low subaerial height of 15–18 m^38^, the potential landslide volume would be very low (Sect. 3.2). This and the fact that volcano and landslide induced tsunamis are characterized by short waves showing high dispersion^44,45^, indicate that the impact of a hypothetical tsunami due to a landslide at Home Reef would likely be very low and local only. Furthermore, it probably would not affect the next populated islands Vava’u and Ha’apai (80 km E of Home Reef), but might have impacts on Home Reef Island itself and the unpopulated Late Island (20 km N) (Fig. 1). A more detailed analysis of tsunami propagation and hazards would require high resolution bathymetry data around the island, which is not currently available.

### Estimation of the lava volume

The TSX data allowed for a detailed measurement of the island’s area over time during the 2022–2024 eruption sequence (Fig. 3). Field observations (from distance) by the Tonga Geological Survey (TGS)^38^ provided additional information about the island’s height above sea level. This information was available during the first eruption phase. By combining our satellite-based island area measurements with the TGS height estimates, we calculated a subaerial lava volume emplaced during the initial eruption phase of ~ 1.11 × 10^6^ m³.

Due to the remote location of Home Reef, no information derived from lava samples is yet available on the composition of the lava emplaced during the 2022–2024 activity. To get an estimate of the lava composition, especially about the silica content, which strongly influences the viscosity of the lava, we applied an inversion of Coppola’s^46^ technique for lava volume estimation from thermal satellite data. This approach directly links the so-called time average discharge rate (TADR) with the VRP measured in MODIS and VIIRS data in an empirical relation and allows an estimate of the erupted lava volume (Sect. 5.2.3). We used nighttime data only to measure the VRP to avoid false effects due to sunglint over the ocean.

**Fig. 10:**
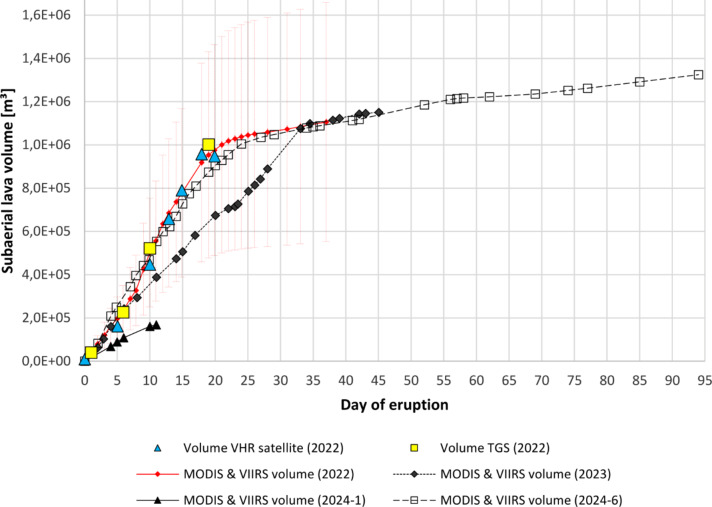
Estimates of the subaerial lava volume. 2022 volume calculation obtained by combining VHR satellite area measurements (blue triangles) (cf. Figures 3) with height information from TGS. Volume estimates of the TGS (yellow squares). Best fit of the MODIS & VIIRS thermal data-based estimates of the lava volume when compared to the VHR based estimates (red diamonds). Volume estimates based on thermal MODIS and VIIRS data assuming same lava composition as for the first eruption: 2023 (black diamonds), January 2024 (black triangle), June-September 2024 (black open square). To improve readability, error bars are shown for the first eruption phase only. The same magnitude of uncertainty is true for the volume estimates of the other eruption phases.

Based on our VHR satellite data lava volume estimates and the ones calculated from the area and height information provided by TGS, a best fit for the inverse of Coppola’s approach could be obtained for silica content of 54.5 wt% (Fig. 10 red line). Due to the ± 50% uncertainty of Coppola’s method regarding the lava volume (Sect. 5.2.3), our derived estimate of silica content is 52–58 wt%, a typical value for andesitic lavas. This is consistent with the silica content of Volcano F caldera samples (54.5 and 59.1 wt% SiO_2_)^15^, the lava composition of Late Volcano (52–58 and 53–63 wt% SiO_2_)^47^ and of Tofua Volcano^48^ (cf. Figure 1). GVP^18^ reports with “dacite” as general information about the previous lava composition of Home Reef imply a slightly higher silica content. Our estimate of the silica content based on thermal satellite data does not replace the need for detailed composition analysis of lava samples in the laboratory once such samples become available, but is used here to assess the erupted volume during the lava flow phases at Home Reef in 2022–2024. The estimate of the absolute lava volumes would slightly change with varying silica content values, however not the relative percentage distribution between the different eruption phases as reported below.

We do not have any independent information regarding the height of the lava flows emplaced during the second to fourth eruption phase. TSX data do not show a step between the lava flows emplaced during the first eruption phase and the new lava flows of the following phases. Therefore, we assume they have similar heights.

Assuming that the lava composition did not change from the first to the following eruption phases, we use the silica content (54.5 wt%) obtained for the first eruption phase to estimate the lava volume emplaced during the second to fourth phase by applying Coppola’s method to MODIS and VIIRS data acquired during those island growth phases (Fig. 10 black lines). The estimated lava volume erupted during the second phase is 1.15 × 10^6^ ± 5.75 × 10^5^ m³, which is about 103% of the volume that erupted during the initial eruption phase. The lava volume estimates for the short-lived January 2024 eruption is 1.68 × 10^5^ ± 8.64 × 10^4^ m³ (15% of the 2022 volume) and for the much longer active fourth phase (94 days) 1.32 × 10^6^ ± 6.60 × 10^5^ m³ (119% of the 2022 volume).

Based on the 9–10 m water depth of the eroded 2006 island (Sect. 1.2), we can estimate an additional submarine part of the lava volume of 50–60% of the subaerial volumes.

## Conclusions

We investigated the 2022–2024 Home Reef eruption from space, starting from the first alerts of the NHI system, using a multi-sensor approach combining satellite observations at different spectral, spatial and temporal resolutions as well as active and passive sensor data allowing for a multi-sensor spatiotemporal analysis of the eruption evolution.

The recent eruption event at Home Reef was characterized by four distinct phases of activity: (i) the first phase started on September 9, 2022, and in the following weeks lava flows generated a new island covering an area (above sea level) of ~ 54,900 m², corresponding to an estimated erupted subaerial volume of ~ 1.11 × 10^6^ m³. We identified, mapped and quantified this eruption in terms of island growth and erosion rate as well as of Volcanic Radiative Power (VRP). (ii) The second eruption phase was longer, but less intense and produced a second lava flow of almost the same volume as the first one, expanding the island to an area of ~ 74,000 m². (iii) The third eruption phase was short-lived and relatively small. (iv) The longest eruptive activity was observed during the fourth phase, which expanded the island’s area to over 122,000 m² (and an estimated total erupted volume of ~ 3.75 × 10^6^ m³ for all phases of the eruption).

The results of our study show that erosion processes during inter-eruption intervals appear to have a strong control on the location of subsequent vents: While the active vent during the first eruption phase was located at the center of the circular island, we observed a migration of the vents that were active during the second, third or fourth eruption phase always to the edge of the remnants of the previous lava flow island where the strongest erosion and therefore the highest load removal occurred in the time period before. The location of the new vent determines the growth direction of the next lava flow. The growth direction may also influence the location of increased instability of the island flanks, which could potentially lead to landslide, which in turn could trigger a local tsunami if a collapse was large enough. However, for the 2022–2024 Home Reef lava flow island, the tsunami hazard could be assumed to be very low and local compared to volcanic islands with much larger potential collapse volumes. Nevertheless, the evolution observed at Home Reef might be used as a model to study the process of volcanic island development together with their related hazards.

## Materials and methods

### Data

Table 1 gives an overview of the satellite data acquired and analyzed over the Home Reef Volcano area. A detailed description is provided below.

**Table 1:** Satellite data acquired and analyzed over the Home Reef Volcano area.

Satellite mission	Sensor type	Bands used	Spatial resolution	Temporal resolution
MODIS	Mid (MIR) and thermal infrared (TIR)	bands 21/22 λ = 3.959 μm, band 31 λ = 11.03 μm	1000 m	4 per day (onboard of the satellites Terra and Aqua)
VIIRS	Mid (MIR) and thermal infrared (TIR)	I4 λ = 3.74 μm, I5 λ = 11.45 μm	375 m	4 per day (onboard of the satellites Soumi NPP and NOAA-20)*
Landsat-8/9 (L8/9)	Multispectral	visible to SWIR	30 m	16 days (one satellite), 8 days with both
Sentinel-2 (S2)	Multispectral	visible to SWIR	10 m and 20 m	5 days (onboard Sentinel-2 A and B)
PlanetScope	Multispectral	visible to NIR	3 m	Daily coverage (constellation of ~ 130 cubesats)
TerraSAR-X	SAR	X-band (3.1 cm)	1.2 m (HS), 0.25 m (ST)	11 days (same orbit)

* VIIRS is also onboard of NOAA-21 (data available since January 17, 2024).

#### High frequency monitoring with low Spatial resolution infrared sensors

All available acquisitions of the sensors MODIS and VIIRS from September 1, 2022, until the end of our observation period on September 17, 2024, were analyzed. Both sensors fly on two satellites each: MODIS on Terra and Aqua, VIIRS on NOAA-20 and the Suomi National Polar-Orbiting Partnership (Suomi NPP). The joint revisit time of MODIS and VIIRS over the Home Reef Volcano area is eight overpasses per day (with NOAA-21 data available since January 2024: twelve overpasses per day). To study thermal anomalies over Home Reef Volcano, their mid (MIR) and thermal infrared (TIR) bands were considered (Table 1)^49,50^. MODIS and VIIRS hotspot data were derived from FIRMS^37^.

#### Monitoring with HR and VHR multispectral sensors

To study the evolution of Home Reef Volcano during the latest island forming eruption period, HR multispectral data acquired by the satellite missions L8/9 and S2 from September 1, 2022, until September 17, 2024, were investigated. Naturally, only clear sky data could be considered for further analysis. The repeat cycle of the Landsat satellites is 16 days each. The joint revisit time of S2A and B is 5 days. All sensors acquire imagery from the visible (and slightly shorter wavelengths, so-called coastal blue) over the NIR towards the SWIR part of the electromagnetic spectrum at 10–20 m (S2) or 30 m (L8/9) spatial resolution.

Seven scenes of atmospherically corrected PS imagery acquired during the eruption phases were available and analyzed: on September 8 and 9 and October 15, 2022, as well as on September 2 and 27, October 17, November 30, 2023. PS data are characterized by 8 bands covering the coastal blue to NIR part at 3 m spatial resolution. PS, operated by Planet Labs, is a constellation of ~ 130 cubesat satellites^31^.

#### Monitoring with VHR SAR sensor

A time series of VHR TSX data from September 20, 2022, until September 17, 2024, were acquired and analyzed. We tracked the evolution of Home Reef Island with time series of (1) HighResolution SpotLight (HS, 1.2 m spatial resolution) and with (2) Starring SpotLight (ST, 0.25 m spatial resolution) data, both with an observation frequency of 11 days in most cases (few with 22 days repeat cycle).

### Methods

#### Analysis of the island evolution by means of multispectral and SAR remote sensing

First, a visual inspection of the satellite data acquired by the multispectral sensors L8/9, S2 and PS was performed to detect volcanic activity such as volcanic ash and water vapor plumes and thermal activity. The following band combinations were used: PS true color band combination 6/4/2 (red λ = 0.665 μm / green λ = 0.565 μm / blue λ = 0.490 μm), S2 MSI false color composite 12/4/2 (SWIR-2 λ = 2.190 μm / red λ = 0.665 μm / blue λ = 0.490 μm), L8/9 OLI/OLI-2 (SWIR-2 λ = 2.20 μm / red λ = 0.665 μm / blue λ = 0.480 μm).

The PS data acquired over the Home Reef Volcano area showed geolocation errors of several meters compared to the S2, L8/9 and TSX data. The geolocation of the PS imagery was corrected by georeferencing the PS data with S2 and or TSX data with the shortest temporal gap to the PS acquisitions.

The spatio-temporal evolution of the newly born Home Reef Island was analyzed by measuring the outline and area of the island in all clear sky multispectral L8/9, S2 and PS images as well as in the TSX HS and ST SAR images. In the beginning of the 2022 eruption, all data were considered for the analysis of the areal development. Later on, after we started the acquisition of VHR TSX data, we concentrated the analysis on these VHR weather independent SAR data. The following parameters were derived from the time series: (1) area of the original lava flow island and (2) area of the entire island including lava flows and sediments; moreover by comparison with the previous measurement following was calculated: (3) stable island area (no change in area), (4) area of island growth / accumulation of sediments, (5) area of island erosion, (6) island area with no change on the surface of the island.

The parameter (6) is based on the interferometric coherence derived from TSX SSC (Single Look Slant Range Complex) data pairs acquired one after the other at the same imaging geometry and polarization, i.e. same pass direction, relative orbit and incidence angle. Precise orbit information (so-called scientific orbit) was considered for accurate co-registration of the data using the SARscape^®^ software implemented in the ENVI^®^ software package. Next, the interferometric coherence was computed, which describes the phase stability between the two SAR acquisitions of the data pair. The coherence reaches from 0 (complete de-correlation) to 1 (stable). We choose a coherence threshold of > 0.5 to identify areas on the island with no change at the surface between the two SAR acquisitions. The coherence is sensitive to small changes not always visible in the radar amplitude. The evolution of the coherence provides information on changes at the surface due to new lava flows or erosion procedures on top of the island. These changes are not visible using the area measurements.

To estimate the emplaced lava volume, we combined the island area measurements by means of the VHR and HR multispectral and SAR data with height estimates reported by TGS by field observations from distance^38^ (cf. Discussion Sect. 3.2).

#### Analysis of the thermal activity of Home Reef Volcano by means of infrared data

To study the thermal activity of Home Reef Volcano once the volcano had grown above sea level, we analyzed NIR and SWIR data from S2 and L8/9 satellites by means of the NHI algorithm, which uses two normalized indices, based on TOA (Top of the Atmosphere) radiances measured at 0.8 μm ($\:{L}_{\text{N}\text{I}\text{R}\:}$), 1.6 μm ($\:{L}_{\text{S}\text{W}\text{I}\text{R}1\:}$) and 2.2 μm ($\:{L}_{\text{S}\text{W}\text{I}\text{R}2\:}$), to map volcanic thermal anomalies by means of daytime L8/9 OLI/OLI-2 and S2 MSI data (Eqs. (1) and (2)).

1 $$\:{\:\:\:NHI}_{SWIR}=\frac{{L}_{\text{S}\text{W}\text{I}\text{R}2\:}-{L}_{\text{S}\text{W}\text{I}\text{R}1\:}}{{L}_{\text{S}\text{W}\text{I}\text{R}2\:}+{L}_{\text{S}\text{W}\text{I}\text{R}1\:}}\:\:\:\:\:$$

2 $$\:{NHI}_{SWNIR}=\frac{{L}_{\text{S}\text{W}\text{I}\text{R}1\:}-{L}_{\text{N}\text{I}\text{R}\:}}{{L}_{\text{S}\text{W}\text{I}\text{R}1\:}+{L}_{\text{N}\text{I}\text{R}\:}}\:\:\:\:\:$$

The algorithm considers pixels showing positive values of one or both the normalized indices as “hot”^51^. The NHI system and tool perform at global scale, under the Google Earth Engine (GEE) environment. The system provides automated notifications about volcanic thermal anomalies, detected over the previous 48 h, whenever its web site is accessed (https://sites.google.com/view/nhi-tool/home-page)^52^. The tool enables time series analysis (since 2013 for L8 OLI data), over the selected volcano, in terms of number of hot pixels, total SWIR radiance and hotspot area. Thermal anomaly maps may be also generated using the system/tool. The latter implements some additional spectral tests to minimize false detection and to account for possible saturation effects in the SWIR bands^53^.

#### Analysis of the erupted lava volume by means of infrared satellite data

Further analysis of thermal activity over Home Reef Volcano was performed by means of MIR data of the sensors MODIS and VIIRS. First, acquisitions of too large scan angles were excluded to assure the reliability of volcanic hotspot detection and to reduce possible distortion effects^10^. Second, following the MIR approach of Wooster et al.^54^, which assumes that the measured heat flux is just related to lava portions having a radiating temperature > 600 K and which is valid for temperatures between 600 and 1500 K, the volcanic radiative power (VRP) was calculated. Wright et al.^55^ confirmed that Wooster’s MIR approach is valid for most active lava bodies. Third, we considered only nighttime acquisitions, to exclude possible false classification of thermal anomalies due to sunglint over the ocean during daytime. Fourth, the total VRP per overflight by MODIS and VIIRS during nighttime was selected. Fifth, the overflight with the maximum total VRP per night was considered.

The approach of Coppola et al.^46^, which directly links the so-called time average discharge rate (TADR) with the VRP in an empirical relation, allows one to estimate the erupted lava volume as described as follows (Eqs. (3), (4)).

3 $$\:TADR=\:\frac{VRP}{{c}_{rad}}$$

with radiant density c_rad_ (in $\:\frac{J}{m^3}$) representing the empirical relationship between radiant and volumetric flux for the analyzed thermal emitting lava and $\:{X}_{{SiO}_{2}}$ describing the silica content (normalized to 100%) of the erupted lava.

4 $$\:{c}_{rad}=\frac{6.45\times\:{10}^{25}}{{\left({X}_{{SiO}_{2}}\right)}^{10.4}}$$

An uncertainty of ± 50% c_rad_ has to be considered because of anticipated significant effects that bulk rheology has on spreading and cooling processes of active lava. Therefore, the TADR was calculated twice: $\:{c}_{{rad}_{min}}=0.5\times\:{c}_{rad}$ and $\:{c}_{{rad}_{max}}=1.5\times\:{c}_{rad}$. Then, the mean of these two calculations was taken to get the final TADR^46^.

An integral analysis of the TADR of sequential satellite acquisitions t_i_ and t_j_ allows one to calculate the erupted lava volume V_t_ (Eq. (5)). The cumulative sum of it results in the total erupted lava volume V (Eq. (6)).

5 $$\:{V}_{t}={\int\:}_{{t}_{i}}^{{t}_{j}}{TADR}_{t}\left(t\right)dt=0.5\times\:\left({t}_{j}-{t}_{i}\right)\times\:\left({TADR}_{{t}_{j}}+{TADR}_{{t}_{i}}\right)$$

6 $$\:V=\:\sum\:{V}_{t}$$

However, as there was no silica content information available (cf. Equation (4)) directly for the 2022 to 2024 eruption phases at Home Reef Volcano nor for previous eruptions at the volcano, we used an inverse of Coppola’s approach as described in detail in the Discussions Sect. 3.2.

## Data Availability

Original satellite data are available via DLR (TerraSAR-X: https://sss.terrasar-x.dlr.de/), NASA (MODIS & VIIRS: https://firms.modaps.eosdis.nasa.gov/, Landsat: https://earthexplorer.usgs.gov/), Planet Labs (PlanetScope: https://www.planet.com/) and ESA (Sentinel-2: https://dataspace.copernicus.eu/). Information derived from the satellite data are available from the corresponding author on request.
